# Adverse Oral Mucosal Reaction to Sublingual Captopril: A Case Report With Exploratory Insights Into AI‐Assisted Clinical Reasoning

**DOI:** 10.1111/scd.70228

**Published:** 2026-07-25

**Authors:** Antônio Roberto Garcia Júnior, Catarina Melquiades Velane, Caroline Akemi Mendes Magario, Paulo Sergio Pina, Carina Domaneschi, Camilla Vieira Esteves

**Affiliations:** ^1^ Department of Stomatology, School of Dentistry University of São Paulo São Paulo São Paulo Brazil; ^2^ Department of Oral Pathology, School of Dentistry University of São Paulo São Paulo São Paulo Brazil

**Keywords:** artificial intelligence, captopril, clinical reasoning, hypertension, mouth mucosa, oral administration, prompt engineering, treatment outcome

## Abstract

**Aims:**

To describe a probable oral mucosal injury associated with the off‐label sublingual administration of captopril in a medically complex patient, and to illustrate the role of structured clinical reasoning in identifying route‐related adverse drug reactions, with exploratory insights into AI‐assisted reasoning.

**Methods and Results:**

A 77‐year‐old patient presented with persistent burning pain and a progressive oral mucosal lesion on the floor of the mouth. Despite appropriate management of local infectious and mechanical factors, symptoms worsened over time. A consistent temporal relationship was observed between lesion exacerbation and repeated sublingual captopril use during hypertensive episodes. Structured clinical reasoning, including iterative causal analysis, supported identification of a probable route‐related adverse drug reaction. Discontinuation of sublingual captopril, combined with topical corticosteroid therapy, resulted in complete resolution of the lesion. An exploratory interaction with a large language model was conducted to examine how structured clinical input may influence the coherence and clinical relevance of AI‐assisted reasoning.

**Conclusion:**

Sublingual administration of captopril may cause localized chemical injury to the oral mucosa, particularly in vulnerable patients with complex medical conditions. Recognition of route‐specific adverse effects is essential to avoid unnecessary interventions and improve patient outcomes. This case also illustrates that AI‐assisted reasoning is highly dependent on the structure and quality of clinical input, supporting its role as a complementary cognitive tool rather than an autonomous diagnostic system.

## 1 Introduction

Hypertensive episodes are a frequent cause of emergency care, particularly among elderly patients with multimorbidity and polypharmacy [1, 2]. Although angiotensin‐converting enzyme inhibitors remain a cornerstone of chronic blood pressure management, the sublingual use of captopril persists in some clinical settings despite the absence of consistent evidence supporting faster or clinically superior blood pressure reduction compared with oral administration [3, 4, 5].

The continued use of sublingual captopril appears to be driven more by clinical habit than by guideline‐based recommendations. While systemic adverse effects of angiotensin‐converting enzyme inhibitors are well established, local adverse reactions related specifically to the route of administration remain underrecognized in the cardiovascular literature. These effects may be clinically relevant, particularly when they exacerbate pain and contribute to sympathetic activation in patients presenting with acute elevations in blood pressure [3, 6].

Oral adverse effects associated with systemic cardiovascular medications, including dysgeusia, burning mouth sensation, lichenoid reactions, and angioedema, are well documented. However, adverse reactions arising from off‐label routes of administration, rather than from the pharmacological class itself, are less frequently reported and may be overlooked in routine clinical practice [7].

The sublingual mucosa is thin, non‐keratinized, and highly vascularized, which facilitates rapid drug absorption but also increases susceptibility to direct chemical injury. Repeated exposure to medications not specifically formulated for sublingual use may result in localized mucosal damage. Failure to recognize such route‐related adverse effects may delay the identification of iatrogenic factors contributing to symptom persistence and recurrent clinical presentations [7, 8].

Despite their clinical relevance, two important gaps persist in the literature. First, local mucosal reactions resulting specifically from off‐label sublingual drug administration remain poorly characterized, with few reported cases documenting a clear causal relationship. Second, the application of structured prompt frameworks to improve AI‐assisted reasoning in complex oral medicine scenarios has not been systematically explored. Whether prompt structure influences the quality of large language model (LLM) output in adverse drug reaction identification remains an open question [9, 10].

This report aims to describe a probable route‐related adverse drug reaction associated with sublingual captopril resulting in oral mucosal injury, and to explore whether the use of the structured COP (Context–Objectives–Persona) prompt framework influences AI‐assisted clinical reasoning in this context.

## 2 Case Description

A 77‐year‐old Black patient presented to an emergency dental clinic with severe burning pain in the floor of the mouth, impairing oral intake and prosthesis use. Her medical history included systemic arterial hypertension, diabetes mellitus, cardiovascular disease, and gastritis. She denied tobacco and alcohol use. At presentation, blood pressure was 168/96 mmHg, and she remained clinically stable.

**FIGURE 1:**
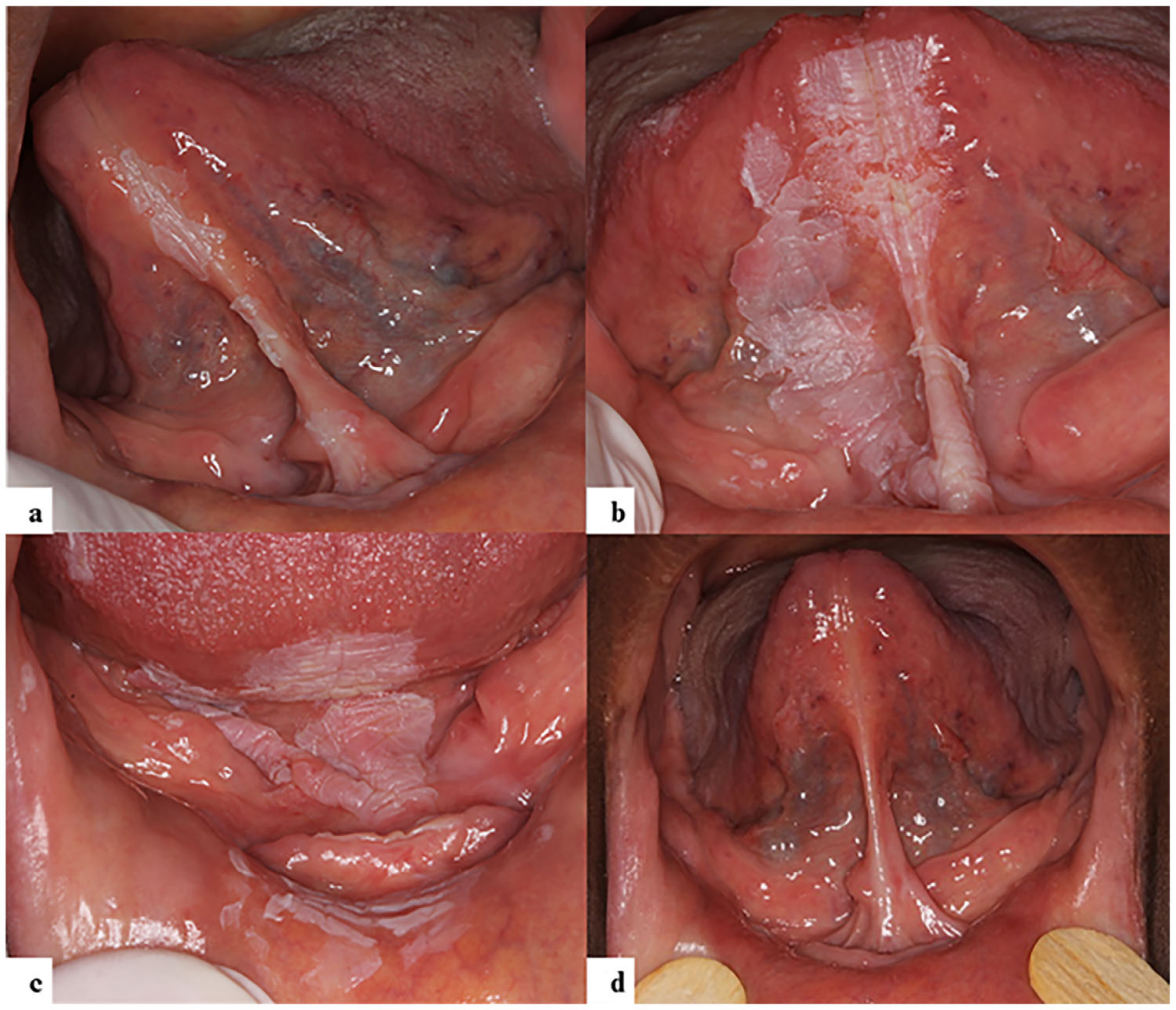
A Intraoral examination revealed a whitish lesion on the floor of the mouth, situated near the sublingual caruncle and midline of the ventral tongue, following the trajectory of the lingual frenulum. The lesion was slightly elevated, measured approximately 2 mm, with a homogeneous surface and defined borders. b, c Lesion progression with expansion across the floor of the mouth and ventral surface of the tongue, rugose surface, epithelial desquamation, and focal erythema. Margins undefined. Persistent cyclical pain despite prior interventions. d After discontinuation of captopril, complete clinical resolution. Mucosa appears normal, without erythema, thickening, or surface alterations.

Intraoral examination revealed an infected ranula and a whitish lesion on the ventral tongue. At this initial stage, marsupialization of the ranula was performed, followed by extraction of two mobile mandibular teeth associated with prosthesis‐related trauma. Although the whitish lesion was already present at the initial visit, it progressively worsened over subsequent consultations, becoming more extensive and well demarcated (Figure 1a).

Despite appropriate local management, including resolution of the ranula, tooth removal, and prosthesis adjustment, the patient continued to experience persistent and worsening pain. She returned repeatedly for emergency care as symptoms did not improve.

Between visits, she presented multiple times with episodes of marked blood pressure elevation associated with orofacial pain. On each occasion, these episodes were managed with sublingual captopril. Following each repeated exposure, the mucosal lesion consistently worsened, progressing to a well‐demarcated lesion consistent with chemical injury (Figure 1b,c). This recurrent pattern suggested a temporal relationship between hypertensive episodes, repeated sublingual drug exposure, and lesion progression.

To support clinical reasoning under conditions of diagnostic uncertainty and competing etiologies, structured approaches were applied. A semiotic framework based on Greimas's semiotic square was used to distinguish essential from accessory clinical elements, allowing initial prioritization of infectious and mechanical factors [11, 12].

However, persistent symptom recurrence despite appropriate interventions prompted a stepwise causal reassessment using an iterative root cause analysis (the “5 Whys” method) [13]. This process clarified the underlying mechanism as follows: (1) the lesion persisted due to ongoing inflammation; (2) inflammation persisted due to repeated local injury; (3) injury resulted from recurrent sublingual exposure to captopril; (4) captopril was administered during episodes of elevated blood pressure; and (5) the association was initially overlooked because findings were attributed to mechanical and prosthetic factors.

This structured reassessment redirected the diagnostic focus from presumed mechanical trauma to a probable route‐related adverse drug reaction. *After discontinuation of sublingual captopril*, combined with topical corticosteroid therapy, marked clinical improvement and complete resolution of the lesion were achieved (Figure 1d).

## 3 AI‐Assisted Clinical Reasoning: Exploratory Use in a Challenging Diagnostic Context

In complex clinical scenarios characterized by diagnostic uncertainty and competing etiological hypotheses, the organization and interpretation of clinical information may represent a significant cognitive challenge. In this context, LLMs have been increasingly explored as tools to support clinical reasoning, not by generating independent diagnoses, but by assisting in the structuring and articulation of available data. In the present case, an exploratory interaction with a LLM (ChatGPT‐5, OpenAI) was conducted to examine how different levels of structure in clinical input could influence the coherence and clinical relevance of AI‐generated reasoning in a diagnostically challenging scenario.

Two input strategies were employed. The first consisted of an unstructured narrative describing the clinical presentation, without explicit temporal organization or hypothesis framing. The second followed a structured approach based on the Context–Objectives–Persona (COP) framework, in which the clinical information was organized chronologically, the diagnostic objective was explicitly defined, and the model was instructed to assume the role of an oral medicine specialist evaluating a potential adverse drug reaction. While the unstructured input generated a broad and non‐prioritized list of possible etiologies, the structured input produced a more coherent reasoning sequence, explicitly integrating the temporal association between sublingual captopril exposure and lesion progression into a plausible causal explanation.

Importantly, the model did not independently establish the diagnosis, but rather reflected and reorganized the clinical reasoning embedded in the structured input, reinforcing its role as a cognitive support tool rather than an autonomous diagnostic system. This exploratory use suggests that LLMs may be particularly useful in challenging cases by helping clinicians externalize and reassess complex causal relationships. However, the observed differences cannot be attributed exclusively to the prompt framework itself, as multiple dimensions of input structure were modified simultaneously, and the analysis was limited to a single case and a single model interaction. Therefore, these findings should be interpreted as illustrative, highlighting the potential role of structured input in AI‐assisted reasoning, while underscoring the need for systematic evaluation in future studies.

## 4 Discussion

This case highlights a probable route‐related adverse drug reaction associated with the sublingual administration of captopril, resulting in localized oral mucosal injury. Although captopril is widely used in the management of hypertensive episodes, its sublingual administration remains an off‐label practice. A 2024 randomized clinical trial and prior meta‐analyses confirm no significant advantage of sublingual over oral captopril in achieving blood pressure reduction during hypertensive urgency, while tolerability is reported to be lower with sublingual use [3, 5]. The direct and repeated contact of captopril with the oral mucosa provides a plausible mechanistic explanation for the well‐demarcated lesion compatible with a chemical burn observed in the present case.

Pain is a recognized physiological stimulus that may increase sympathetic activity and transiently elevate blood pressure, particularly in elderly patients [6]. In this context, the local adverse reaction to sublingual captopril may itself have contributed to transient increases in blood pressure, perpetuating a cycle of pain, hypertension, and further drug exposure. The continued prescription of sublingual captopril despite limited evidence of net clinical benefit reinforces the need to reassess empirically adopted practices in cardiovascular and emergency care.

The diagnostic process required integration of multiple clinical elements under conditions of uncertainty. Initial interpretation prioritized mechanical and infectious factors, supported by findings related to prosthetic trauma and ranula. Persistence of symptoms prompted reassessment of causal relationships.

Greimas's semiotic square provided a structural framework to distinguish essential from accessory findings, reducing the influence of misleading associations [11, 12]. The “5 Whys” method enabled iterative reconstruction of the causal chain, linking persistent inflammation to repeated chemical exposure and, ultimately, to the off‐label use of sublingual captopril [13]. Anamnesis played a central role by establishing the temporal association between drug administration and symptom exacerbation, supporting the transition from initial pattern recognition to structured causal reassessment.

While prior studies have demonstrated that structured prompting can improve LLM performance [9, 10, 14], these findings are primarily based on controlled experimental settings. In contrast, the present report illustrates how, in a real‐world diagnostic scenario, AI output appears to reflect the structure of clinician‐generated reasoning rather than independently enhancing it.

Important limitations must be considered. The model did not independently explore alternative competing diagnoses and relied entirely on the structure and quality of the input provided. This confirms that AI does not autonomously interpret clinical signs, but reflects patterns derived from the data it receives. Its outputs are inherently dependent on clinician‐guided framing and should be understood as extensions of organized clinical reasoning rather than independent sources of diagnostic inference [9, 10].

Taken together, this case suggests that structured prompting, including approaches such as COP, may contribute to more coherent and clinically relevant AI‐assisted reasoning outputs in complex diagnostic scenarios. Further investigation is warranted to define the optimal integration of such tools in adverse drug reaction assessment and oral medicine clinical practice.

## 5 Conclusion

The off‐label sublingual use of captopril in hypertensive episodes does not provide a clearly established clinical advantage and may expose patients to preventable local adverse effects. Reconsideration of this practice and greater adherence to evidence‐based approaches to blood pressure management are particularly warranted in elderly and medically complex patients.

This case highlights the importance of recognizing route‐specific adverse reactions as potential contributors to symptom persistence and clinical instability. It also demonstrates that AI‐assisted clinical reasoning is highly dependent on the structure and completeness of clinical input: the COP‐structured prompt enabled the model to reproduce a coherent causal reasoning pathway aligned with the clinical diagnosis, while unstructured input yielded non‐specific responses. These findings suggest that structured prompt frameworks may serve as practical cognitive support tools in complex clinical scenarios, provided they are guided by clinician expertise rather than used as substitutes for clinical judgment.

## Funding

This study was supported by the São Paulo Research Foundation (FAPESP) process number (2025/22650‐4) through an undergraduate research scholarship.

## Ethics Statement

All procedures performed in studies involving human participants were in accordance with the ethical standards of the institutional and national research committee and with the 1964 Helsinki declaration and its later amendments or comparable ethical standards. The study was approved by the Research Ethics Committee of the School of Dentistry, University of São Paulo (FOUSP) under CAAE number 94936525.9.0000.0075.

## Informed Consent

Written informed consent was obtained from the patient for publication of this case report, including accompanying images.

## Conflicts of Interest

The authors declare no conflicts of interest.

## Learning Objectives

To highlight a clinically relevant oral adverse effect associated with off‐label sublingual captopril use and to emphasize the importance of recognizing route‐related drug reactions in medically complex patients.

## Declaration of generative AI use

During the preparation of this work, the authors used ChatGPT (OpenAI) in order to support language refinement, improve clarity and organization of the text, and assist in structuring sections related to the discussion of artificial intelligence–assisted reasoning. The tool was also used in an exploratory manner within the study to assess its ability to reproduce structured clinical reasoning based on the information provided. After using this tool, the authors carefully reviewed, edited, and validated all content as needed and take full responsibility for the accuracy, integrity, and originality of the final manuscript.

## Data Availability

The data generated and/or analyzed during the current study are available and can be requested from the corresponding author.
